# Effects of Hydrological Regime on Foliar Decomposition and Nutrient Release in the Riparian Zone of the Three Gorges Reservoir, China

**DOI:** 10.3389/fpls.2021.661865

**Published:** 2021-05-26

**Authors:** Zhangting Chen, Muhammad Arif, Chaoying Wang, Xuemei Chen, Changxiao Li

**Affiliations:** ^1^Key Laboratory of Eco-Environments in the Three Gorges Reservoir Region (Ministry of Education), State Cultivation Base of Eco-Agriculture for Southwest Mountainous Land, College of Life Sciences, Southwest University, Chongqing, China; ^2^School of Tourism Management, Guilin Tourism University, Guilin, China; ^3^Chongqing City Management College, Chongqing, China

**Keywords:** foliar decomposition, nutrient release, hydrological regime, mixed species, riparian zone, Three Gorges Reservoir

## Abstract

Foliar decomposition has significant effects on nutrient cycling and the productivity of riparian ecosystems, but studies on the impact of related hydrological dynamics have been lacking. Here, the litterbag method was carried out to compare decomposition and nutrient release characteristics *in situ*, including three foliage types [two single-species treatments using *Taxodium distichum* (L.) Rich., *Salix matsudana* Koidz., or a mixture with equal proportions of leaf mass], three flooding depths (unflooded, shallow flooding, and deep flooding), two hydrodynamic processes (continuous flooding and flooded-to-unflooded hydrological processes), and one hydrological cycle (1 year) in the riparian zone of the Three Gorges Reservoir. The results showed that both hydrological processes significantly promoted foliage decomposition, and all foliage types decomposed the fastest in a shallow flooding environment (*P* < 0.05). The mixed-species samples decomposed most quickly in the flooded hydrological process in the first half of the year and the unflooded hydrological process in the second half of the year. Flooding also significantly promoted the release of nutrients (*P* < 0.05). Mixed-species samples had the fastest release rates of carbon and nutrients in the flooded hydrological process in the first half of the year and the unflooded hydrological process in the second half of the year. Foliage decomposition was also closely related to environmental factors, such as water depth, temperature, and hydrological processes. Our research clarified the material cycling and energy flow process of the riparian ecosystem in the Three Gorges Reservoir area. It also provided a new reference for further understanding of foliage decomposition and nutrient release under different hydrological environments.

## Introduction

As the largest hydroelectric power project in human history, the Three Gorges Reservoir (TGR) has always received widespread attention (Chen et al., 2020a,b; Arif et al., 2021; He et al., 2021; Zheng et al., 2021). When compared with other reservoirs, the new hydrologic regime of the TGR reverses the variation of the natural dry/flood pattern (rising in winter and falling in summer), with a large fluctuation range (145–175 m a.s.l., up to 30 m) and a prolonged flooding duration each year (Ye et al., 2011; Li et al., 2013; Xu et al., 2013; Yuan et al., 2013; Holbach et al., 2014). This dramatically alters the conditions of riparian ecosystems and results in the formation of a reservoir water-level fluctuation zone (i.e., seasonal fluctuations causing the land to be periodically flooded and dried, and the formation of an alternating dry and wet land–water transition zone, which is defined as the area between the normal water level) (Zhang and Lou, 2011; Yang et al., 2012; Zhang, 2018; Arif et al., 2020). Therefore, the riparian ecosystem in the TGR has both abundant resources in the terrestrial ecosystem and periodic hydrological fluctuations in the aquatic ecosystem, creating a huge area with a unique biogeochemical function. Moreover, riparian ecosystems, as centers of biodiversity and links between terrestrial and aquatic systems, also play an important role in material circulation, energy flow, and maintaining the ecological structure and functional balance (Nilsson and Svedmark, 2002; New and Xie, 2008; González et al., 2017).

**FIGURE 1:**
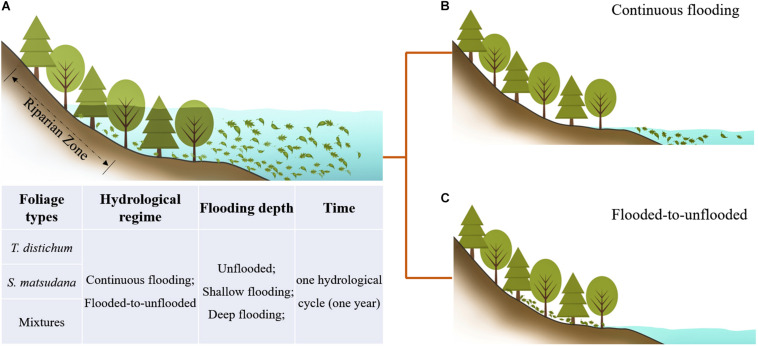
The decomposition process of the leaves of dominant tree species under hydrological regimes in the riparian zone. **(A)** The water level rises, and the leaves undergo flooded decomposition. **(B,C)** The water level drops, and the leaves continue to undergo flooded decomposition or unflooded decomposition.

Due to the unique hydrological regime in the TGR area, the plants within have a special growth pattern. When the water level drops in spring, the trees begin to recover and reach the peak of growth in summer. As the water level rises in autumn, the trees are flooded continuously in a cycle that repeats each year. It is worth noting that when the water level rises, the leaves of the trees are inevitably submerged and then decompose; when the water level drops half a year later, the leaves that have not been completely decomposed may flow downstream with the water and continue to be decomposed in the waterbody, or they may stay on the riparian land and undergo dry condition decomposition. Therefore, with the fluctuation of the water level in the TGR area, the leaves of these plants are subject to periodic continuous flooding or a decomposition environment that alternates between wet–dry (Figure 1). Similarly, when the level rises, the leaves of different tree species are submerged simultaneously and jointly complete the entire process of decomposition, which is what we usually call mixed decomposition. Most studies on different foliage types compared with the decomposition of a single species, which cannot fully and accurately reflect the actual situation of a natural ecosystem. Studies of mixed-species samples have mostly been carried out in terrestrial or aquatic environments independently; a few studies have addressed the decomposition of mixed-species samples in a region that has both aquatic and terrestrial characteristics in the same environment. The effects of hydrological dynamics on foliage decomposition remain unclear, especially in a riparian zone of the TGR area with such a huge land–water interlaced area. This is not conducive to our understanding of the material cycle and energy flow process of the riparian ecosystem.

Foliage decomposition in streams and rivers is a vital process linking ecosystem nutrient cycling, energy transfer, and trophic interactions (Lecerf et al., 2007; Kominoski et al., 2011; Lidman et al., 2017). Many studies have shown that the decomposition of leaves is related to the environmental conditions around them and their physical and chemical properties, such as leaf quality, flooding depth, temperature, nutrient content, and dissolved oxygen (Kominoski et al., 2007; Lecerf et al., 2007, 2011; Ferreira and Chauvet, 2011). Among them, the depth of flooding is an important extrinsic environmental factor that controls foliage decomposition and nutrient release (Sun et al., 2012; Zhang et al., 2019). The influence of water depth on decomposition is mainly reflected in that with the change in water depth, water environmental conditions, such as temperature, dissolved oxygen, redox conditions, and illumination intensity will change, which may further affect the activity of decomposing microorganisms and, thus, affect leaf decomposition (Wallis and Raulings, 2011; Xie et al., 2016). One study has shown that the decomposition rate of leaves decreases with the increase in water depth (Xie et al., 2016), but other studies have shown that water depth has no significant effect on the decomposition rate of leaves (Wright et al., 2013; Arroita et al., 2015). It can be seen that the effect of water depth on the decomposition rate of plants will be different due to changes in species, the decomposition of the microenvironment (water flooding depth), and other changes. In addition, the alternating wet–dry environment also has a certain impact on the decomposition of leaves, mainly due to the extent and duration of flooding (Anderson and Smith, 2002; Capps et al., 2011; Sun et al., 2012; Yu et al., 2020). However, a few studies have addressed the decomposition of different woody plants under different flooding depths and wet–dry environments.

Besides being affected by environmental factors, litter decomposition rates also depend significantly on the characteristics of the litter (Makkonen et al., 2012; Xie et al., 2017; Hoeber et al., 2020). Generally, leaves with a small number of structural macromolecules (lignin and cellulose) and higher nitrogen (N) and phosphorus (P) content are conducive to the colonization of microorganisms, and their decomposition rate is usually higher (Kalburtji et al., 1999; Das et al., 2008; Zhang et al., 2014; Alvim et al., 2015). Some studies have also found that the decomposition rate of soft green leaves with a large surface area is higher than that of hard coniferous litters (Fonte and Schowalter, 2004; Li et al., 2009; Wang et al., 2016). The release dynamics of N and P in the foliage was also the key link of nutrient flow in soil–water (Zhang et al., 2016). To identify the dominant energy pathways and nutrient dynamics in the vast transition zone between terrestrial/aquatic ecosystems of the riparian area, it is imperative to investigate the processing of foliage decomposition and organic matter in such a huge area of the TGR.

According to the actual hydrological conditions of the TGR area, we set up a field decomposition experiment of one hydrological cycle (1 year), including two hydrological regimes (continuous flooding and flooded-to-unflooded hydrological processes), three flooding depths (unflooded, shallow flooding, and deep flooding), and two dominant afforestation tree species [*Taxodium distichum* (L.) Rich., and *Salix matsudana* Koidz.] and their mixtures with equal proportions representing the riparian zone of the TGR. The objectives of this study were to (1) investigate the decomposition processes and rates of the dominant plant species in the riparian zone of the TGR under the two hydrological regimes; and (2) compare the nutrient release of single and mixed-species samples under different flooding depths, and clarify the material cycling and energy flow processes of the riparian ecosystem in the TGR area, providing a new reference for further understanding the decomposition process of leaves and the dynamic characteristics of nutrient release in different hydrological environments.

## Materials and Methods

### Study Site

The study was conducted at a revegetation demonstration site of the Ruxi watershed of Zhong County, Chongqing Municipality (N30°24′16′′ and E108°08′03′′), near the riparian zone of the TGR. After the TGR reached an average water storage level of 175 m in 2010, a very large area of land–water ecotone formed, covering an area of nearly 350 km^2^ (Yangtze River Water Resources Commission, 1999; Wang, 2003; Li et al., 2013; Ren et al., 2016).

Considering the tolerance limit of trees (poor survival below 165 m) and the safety of the river channel, all trees are planted within 165–175 m of the upper part of the riparian zone in a mixed planting with row spacing of 1 m × 1 m. The trees of the study area were dominated by *Taxodium ascendens* Brongn, *Taxodium distichum* (L.) Rich., and *Salix matsudana* Koidz. The Ruxi River Basin belongs to the center of the TGR area and is also an important shipping channel. In order to ensure the safety, accuracy, and operability of the experiment, the decomposition experiment site was selected in an artificial reservoir connected to the Ruxi River (with an area of more than 700 m^2^). The reservoir and Ruxi River are separated by only one artificial dam, and the water of the reservoir flows directly to the Ruxi River. This subtropical southeast monsoon region experiences a humid (relative air humidity of 80%) and a mountainous climate with a mean annual temperature of 18.2°C. The annual sunshine time is 1,327.5 h, and the number of frost-free days is 341 days. Soils are dominated by purple soil.

### Experimental Design

According to the actual hydrological conditions of the TGR, a field foliage decomposition experiment using a litterbag method lasted for a whole year, which is one hydrological cycle, coinciding with the time when the water level rises in the riparian zone of the TGR. Because *T. distichum* and *T. ascendens* belong to the same genus and have many similar features, we chose the coniferous species *T. distichum* and the broad-leaved species *S. matsudana* for the experiments; the mixture used equal proportions of leaf mass from these two species. Trees at different elevations have different degrees of flooding. In order to analyze the nutrient release of plants in different environments, we set up three flooding depth treatments, 0.5 m to represent shallow flooding (SF) and 5 m to represent deep flooding (DF), as well as an unflooded environment as a control (CK). The litterbags of the samples were then randomly divided into five experimental treatments of CK, SF, DF, SF-CK, and DF-CK (CK, unflooded; SF, 0.5 m shallow flooding; DF, 5-m-deep flooding; SF-CK, first half a year SF followed by half a year CK; and DF-CK, first half a year DF followed by half a year CK, relative to the water surface), which simulated the decomposition environment of continuous flooding or flooded-to-unflooded hydrological processes in the TGR area (Figure 1). The SF-CK and DF-CK treatments are referred to collectively as the flooded-to-unflooded treatments below. In September 2017, 15 g of single species of fresh leaves of *T. distichum*, 15 g of single species of fresh leaves of *S. matsudana*, and a mixture of the two samples (*T. distichum* + *S. matsudana*) of equal mass (7.5 + 7.5 g) were prepared and placed in each nylon litterbag (20 cm × 20 cm with a 0.25-mm mesh size). Ten sampling dates, four replications, and a total of 516 samples were selected to monitor the effects of hydrological regimes on foliage decomposition and nutrient release. To assess the initial dried weights, an additional 12 samples of each foliage type combinations were prepared. We placed 120 litterbags in the field (CK); 360 litterbags were placed at SF and DF of the reservoir on September 25, 2017; 6 months later, 120 samples of them were taken from the water and placed on the CK next to the reservoir to experience unflooded decomposition. The other samples remained in the water until the experiment ended for a total of 1 year. After that, sediment or invertebrates were removed from the litterbags at each sampling time; the retrieved samples were oven-dried at 65°C for 48 h to determine dry mass and nutrient concentrations. During the decomposition period, the air and water temperature of the sample site were concurrently recorded at each sampling date using a Hydrolab DS5 water quality multiparameter monitor (Hydrolab-Hach Corp., Loveland, CO, United States) (Table 1).

**TABLE 1:** The average, maximum, and minimum of air temperature, water temperature, dissolved oxygen, and water conductivity during the experimental period.

**Water treatments**	**T (°C)**			**DO (mg⋅L^–1^)**			**WC (μS⋅m^–1^)**		
	**Ave.**	**Maximum**	**Minimum**	**Ave.**	**Maximum**	**Minimum**	**Ave.**	**Maximum**	**Minimum**
CK	23.6	36.7	11.4				409.3	478	374.3
SF	21.6	36.6	9.0	15.2	21.8	11.3	406.2	489.6	325.4
DF	15.7	22.2?	9.1	5.7	9.8	3.4	402.2	490.2	301.1

*CK, unflooded; SF, shallow flooding; DF, deep flooding*.

### Statistical Analysis

The negative exponential decay model proposed by Olson (1963) was used to fit the leaf litter decomposition model:

*y* = ae^–*kt*^,

where *a* is the fitting parameter, *k* is the decomposition coefficient of the litter, and *t* is the decomposition time (a).

The dry mass remaining (MR) of foliage was measured using the method of Xie et al. (2017):

MR (%) = *W_*t*__/_W_0_* × 100%.

The nutrient accumulation index (NAI) of each element during foliage decomposition was calculated using the method of Chen et al. (2017):

NAI = (*W*_*t*_ × *X*_*t*_)/(*W*_0_ × *X*_0_) × 100%,

where *W*_*t*_ is the remaining mass at time *t* (g), *X*_*t*_ is the nutrient concentration at time *t* (g/kg), *W*_0_ is the initial dry mass (g), and *X*_0_ is the initial nutrient concentration (g/kg).

The effects of foliage types, experimental treatment, and decomposition time on the mass and nutrient remaining were revealed by repeat measurements of analysis of variance (ANOVA). If the data did not satisfy the Mauchly sphericity assumption, the more robust Greenhouse–Geisser results for degrees of freedom after correction were used. We used univariate regression analyses with the mass remaining as the response variable and the environment physicochemistry as the predictor variable to explore their relationships. To explore the influence of the hydrological regimes among decomposition periods and the mass remaining and nutrient release during decomposition periods for each foliage type in a given environment, we used a one-way ANOVA to explicitly assess the effects of the foliage types and decomposition period. The exponential regression was used to fit the index of mass remaining and nutrient remaining among experimental treatments to decomposition time. Where the ANOVA results were significant at *P* < 0.05, differences among means were determined using Duncan’s honestly significant difference test. The statistical analyses were carried out using the SPSS 22.0 (SPSS Inc., Chicago, IL, United States) for Microsoft Windows.

## Results

### Foliage Decomposition Under Different Hydrological Conditions

**FIGURE 2:**
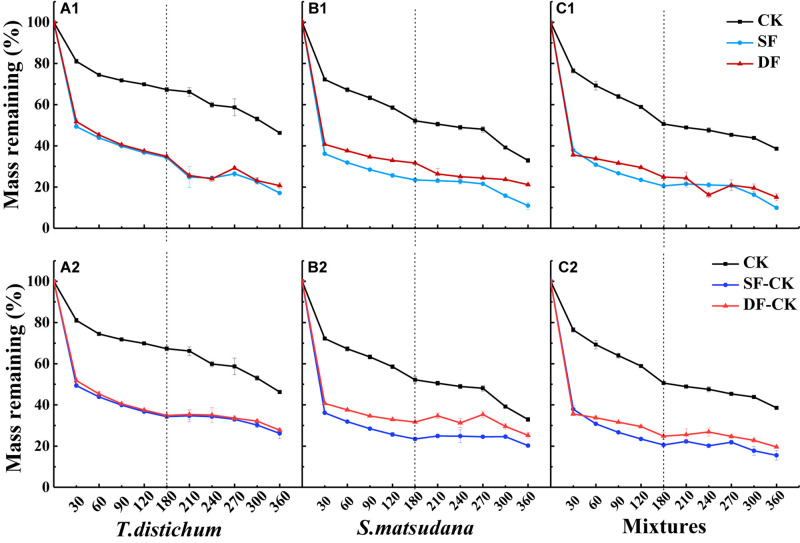
The percentage of mass remaining of leaves of *Taxodium distichum* **(A1,A2)**, *Salix matsudana* **(B1,B2)**, and their mixtures **(C1,C2)** under different hydrological regimes during the decomposition. CK, unflooded; SF, shallow flooding; DF, deep flooding; SF-CK, first half a year SF followed by half a year CK; DF-CK, first half a year DF followed by half a year CK.

The mass remaining (MR) on the foliage types, decomposition time, experimental treatment, and their interactions were very significant (*p* < 0.001; Table 2). The MR decreased significantly with the increase in decomposition time (*p* < 0.05), and the decomposition of all foliage types in each experimental treatment showed the fastest mass loss at the beginning of the decomposition, followed by a gradual slowing down (Figure 2). During the decomposition process in the first half of the year (0–180 days), the leaves of *T. distichum*, *S. matsudana*, and their mixtures decomposed much faster in the flooded environment (both SF and DF) than in the CK (*p* < 0.05) (Figure 2). Regardless of the experimental treatment, the decomposition rate of the mixed species was always the fastest in the first half of the year, and the decomposition rate (*k*′, g⋅g^–1^⋅day^–1^) of CK, SF, and DF reached 3.48, 7.49, and 6.04, respectively (Table 3). Then during the decomposition process in the second half of the year (180–360 days), the experimental treatment was divided into two hydrological processes: continuous flooding decomposition and flooded-to-unflooded decomposition. As in the first half of the year, the decomposition rate of the two hydrological processes was significantly greater than that of CK (*p* < 0.05); that is, flooding significantly promoted foliage decomposition (Figure 2). The decomposition rate of each foliage type that continued under flooding decomposition (SF and DF) was significantly greater than that of the dry decomposition environment (SF-CK and DF-CK) (*p* < 0.05). Except for the CK and SF environments, the decomposition rates (*k*′, g⋅g^–1^⋅day^–1^) of the mixed-species in the second half of the year were also the fastest, reached 2.52, 1.96, and 1.38 in the DF, SF-CK, and DF-CK environments, respectively (Table 3).

**TABLE 2:** Analysis of variance (ANOVA) results for the foliage and TOC, TN, and TP remaining during the decomposition process.

**Source of variation**	***F*-value**			
	**MR**	**CR**	**NR**	**PR**
Foliage type	554.50	158.06	58.04	18.59
Experimental treatment	2,020.25	1,115.86	568.88	1,630.63
Time	4,419.01	2,727.58	946.27	2,640.88
Foliage type × experimental treatment	15.69	8.57	6.75	45.80
Foliage type × time	12.91	9.45	3.06	8.50
Time × experimental treatment	44.38	24.21	19.98	53.10
Foliage type × time × experimental treatment	2.92	3.11	1.86	2.85

*MR, mass remaining; CR, total carbon remaining; NR, nitrogen remaining; PR, phosphorus remaining; all P < 0.001*.

**TABLE 3:** Changes in decomposition rate (*k*′, g⋅g^–1^⋅day^–1^) of leaves of *Taxodium distichum* (A), *Salix matsudana* (B), and their mixtures (C) under different experimental treatments.

**Foliage type**	**Experimental treatment**	**First (0–180 days)**		**Second (180–360 days)**	
		***k*′ (g⋅g^–1^⋅dy^–1^)**	***R*^2^**	***k*′ (g⋅g^–1^⋅day^–1^)**	***R*^2^**
A	CK	1.95	0.73	2.15	0.80
	SF	4.97	0.68	3.00	0.48
	DF	4.99	0.70	2.30	0.51
	SF-CK			1.63	0.47
	DF-CK			1.28	0.37
B	CK	3.18	0.84	2.65	0.84
	SF	6.60	0.64	4.54	0.70
	DF	5.02	0.56	1.91	0.60
	SF-CK			0.76	0.11
	DF-CK			1.38	0.35
C	CK	3.48	0.91	1.48	0.90
	SF	7.49	0.71	4.04	0.69
	DF	6.04	0.61	2.52	0.32
	SF-CK			1.96	0.33
	DF-CK			1.38	0.29

*First: the first half of the year (0–180 days); second: the second half of the year (180–360 days); k′, k × 10^3^. CK, unflooded; SF, shallow flooding; DF, deep flooding; SF-CK, first half a year SF followed by half a year CK; DF-CK, first half a year DF followed by half a year CK*.

The amount of foliage mass remaining was significantly related to many measured environmental factors, but there were differences between different experimental treatments (Table 4). In the first half of the decomposition period, the mass remaining of all foliage types were significantly positively correlated with temperature, while the electrical conductivity, pH, and dissolved oxygen in the SF and DF environments were significantly negatively correlated with the mass remaining. In the second half of the decomposition period, under continuous flooding of the SF and DF environments, the mass remaining of all foliage types were significantly positively correlated with electrical conductivity and dissolved oxygen. The mass remaining of the leaves of *T. distichum* in the SF and DF environments, and leaves of *S. matsudana* and mixtures in the DF environments were also significantly positively correlated with pH. However, in the SF-CK and DF-CK environments, the mass remaining of each foliage type was not significantly correlated with environmental factors (Table 4).

**TABLE 4:** *F*-value and determination coefficient (*R*^2^, in parenthesis) for the regression analyses with foliage mass remaining rate (percent initial mass/month) as response variables and soil or water physiochemistry as predictor variables for the different experimental treatments over the 1-year experiment.

**Foliage types**	**Experimental treatment**	**First (0–180 days)**			
		**T (°C)**	**WC (μS⋅m^–1^)**	**pH**	**DO (mg⋅L^–1^)**
A	CK	+18.81***0.461	0.26(0.012)	+5.02*(0.186)	
	SF	+19.05***(0.464)	−38.5***(0.636)	−15.97**(0.421)	−29.07***(0.569)
	DF	+18.15***(0.452)	−44.07***(0.667)	−66.12***(0.750)	−22.83***(0.509)
B	CK	+12.91**(0.370)	0.96(0.042)	3.83(0.148)	
	SF	+18.55***(0.457)	−32.1***(0.593)	−14.13**(0.391)	−23.95***(0.521)
	DF	+12.38**(0.360)	−39.2***(0.641)	−42.16***(0.657)	−14.92**(0.404)
C	CK	+11.98**(0.352)	1.63(0.069)	3.37(0.133)	
	SF	+19.21***(0.466)	−35.99***(0.621)	−15.08**(0.407)	−27.3***(0.554)
	DF	+10.85**(0.330)	−36.44***(0.624)	−38.53***(0.637)	−15.09**(0.407)

**Foliage types**	**Experimental treatment**	**Second (180–360 days)**			
		**T(°C)**	**WC (μS⋅m^–1^)**	**pH**	**DO (mg⋅L^–1^)**

A	CK	2.92(0.117)	0.03(0.001)	0.30(0.013)	
	SF	0.33(0.015)	+12.12**(0.355)	+4.56*(0.172)	+12.44**(0.361)
	DF	3.25(0.129)	+14.13**(0.391)	+18.87***(0.462)	+10.82**(0.330)
	SF-CK	0.06(0.003)	0.37(0.016)	0.05(0.002)	
	DF-CK	0.15(0.007)	0.1(0.005)	0.21(0.01)	
B	CK	2.02(0.084)	0.17(0.008)	<0.001	
	SF	0.36(0.016)	+17.09***(0.437)	0.108(0.005)	+13.89**(0.387)
	DF	−6.16*(0.219)	+26.13***(0.543)	+12.53**(0.358)	+22.7***(0.508)
	SF-CK	1.33(0.057)	0.03(0.001)	1.85(0.078)	0.13(0.006)
	DF-CK	0.04(0.002)	<0.01	0.55(0.024)	3.12(0.124)
C	CK	2.42(0.099)	<0.001	0.05(0.002)	
	SF	0.03(0.001)	+9.48**(0.301)	0.05(0.002)	+7.64*(0.258)
	DF	1.36(0.058)	+7.65*(0.258)	+9.14**(0.293)	+6.23*(0.221)
	SF-CK	<0.001	0.169(0.007)	0.9(0.039)	
	DF-CK	0.03(0.001)	0.44(0.019)	0.19(0.008)	

*Significantly positive and negative relationships are indicated by (+) and (−), respectively*. A: Taxodium distichum; B: Salix matsudana; C: Mixtures. First: the first half of the year (0–180 days); Second: the second half of the year (180–360 days); T, temperature; WC, water conductivity; DO, dissolved oxygen. *P < 0.05, **P < 0.01, ***P < 0.001.

### Nutrient Release Under Different Hydrological Conditions

The nutrients remaining in the foliage types, decomposition time, experimental treatments, and their interactions were very significant (*p* < 0.001; Table 2). During the decomposition process in the first half of the year (0–180 days), the TOC and TN release rate of mixed species was the fastest under CK and SF environments, while in the DF environment, the *T. distichum* was the fastest. The TP release rate of the three foliage types was faster than that of TOC and TN. In the CK and DF environments, the TP release rate (*k*′, g⋅g^–1^⋅day^–1^) of *T. distichum* was the fastest, reaching 4.04 and 8.62, respectively, while under SF environment, the TP release rate of mixed species was the fastest, reaching 9.21 (Table 5). Then in the second half of the decomposition period

**TABLE 5:** Changes in C, N, and P release rates (*k*′, g⋅g^–1^⋅day^–1^) of leaves of *Taxodium distichum* (A), *Salix matsudana* (B), and their mixtures (C) under different experimental treatments.

**Foliage type**	**Experimental treatment**	**TOC**		**TN**		**TP**	
	
				**First (0–180 days)**			
	
		***K*′**	***R*^2^**	***k*′**	***R*^2^**	***k*′**	***R*^2^**
A	CK	1.84	0.65	0.72	0.24	4.04	0.63
	SF	4.67	0.72	4.11	0.76	7.19	0.62
	DF	7.15	0.76	6.64	0.83	8.62	0.71
B	CK	2.79	0.71	1.11	0.43	2.10	0.50
	SF	6.53	0.73	5.28	0.76	8.39	0.42
	DF	6.19	0.61	5.32	0.68	7.82	0.44
C	CK	3.24	0.81	1.22	0.44	2.73	0.40
	SF	7.34	0.78	5.75	0.77	9.21	0.59
	DF	6.85	0.69	5.66	0.67	7.94	0.52

**Foliage type**	**Experimental treatment**	**TOC**		**TN**		**TP**	
		
				**Second (180–360 days)**			
	
		***k*′**	***R*^2^**	***k*′**	***R*^2^**	***k*′**	***R*^2^**

A	CK	4.84	0.87	3.71	0.77	7.05	0.86
	SF	4.51	0.54	2.45	0.29	4.01	0.64
	DF	2.52	0.47	2.06	0.42	4.93	0.78
	SF-CK	4.91	0.66	2.86	0.52	3.09	0.46
	DF-CK	4.23	0.42	2.48	0.25	3.54	0.42
B	CK	3.59	0.62	3.20	0.77	6.06	0.89
	SF	6.77	0.72	6.49	0.74	2.73	0.51
	DF	1.71	0.35	1.84	0.21	4.61	0.84
	SF-CK	2.53	0.35	0.51	0.02	3.60	0.64
	DF-CK	3.35	0.32	2.25	0.16	3.85	0.61
C	CK	1.55	0.39	3.38	0.68	3.42	0.44
	SF	4.76	0.74	4.97	0.81	6.66	0.73
	DF	2.85	0.37	4.25	0.46	5.83	0.71
	SF-CK	4.76	0.71	3.09	0.46	4.51	0.49
	DF-CK	4.83	0.57	3.54	0.42	4.65	0.70

*First: the first half of the year (0–180 days); second: the second half of the year (180–360 days); k′: k × 10^3^; CK, unflooded; SF, shallow flooding; DF, deep flooding; SF-CK, first half a year SF followed by half a year CK; DF-CK, first half a year DF followed by half a year CK*.

(180–360 days), the TOC (*k*′, g⋅g^–1^⋅day^–1^) release rates of the CK and SF-CK environments of *T. distichum* and the SF environment of *S. matsudana* were the highest. The TOC release rates of mixed species in the other DF and DF-CK environments were the highest, reaching 2.85 and 4.83. The TN (*k*′, g⋅g^–1^⋅day^–1^) release rates of the CK environment of *T. distichum* and the SF environment of *S. matsudana* were the highest, and the TN release rates of mixed species in the other DF, SF-CK, and DF-CK environments were the highest, reaching 4.25, 3.09, and 3.54. The TP (*k*′, g⋅g^–1^⋅day^–1^) release rate of the CK environment of *T. distichum* was the highest, and the TP release rates of mixed species in the other SF, DF, SF-CK, and DF-CK environments were the highest, reaching 6.66, 5.83, 4.51, and 4.65 (Table 5).

### Nutrient Accumulation Index During Foliage Decomposition

**FIGURE 3:**
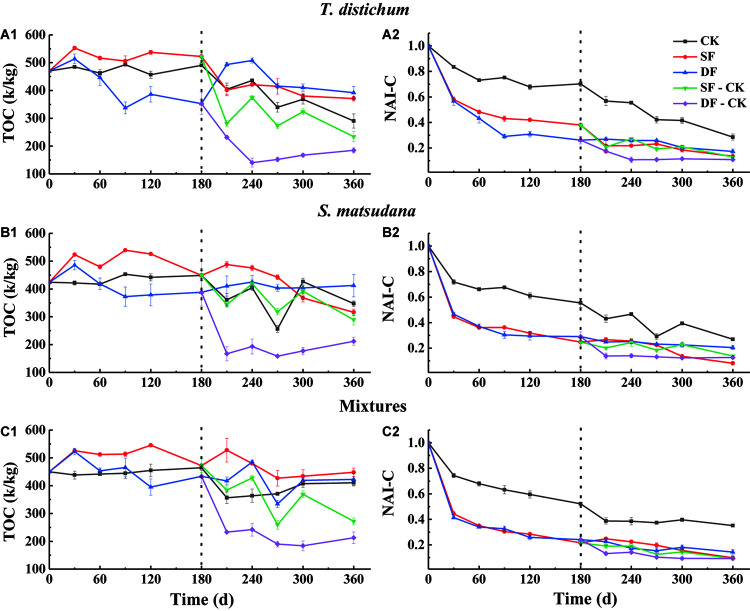
The variation of total carbon concentration **(A1–C1)** and NAI-C index **(A2–C2)** during the process of foliage decomposition. CK, unflooded; SF, shallow flooding; DF, deep flooding; SF-CK, first half a year SF followed by half a year CK; DF-CK, first half a year DF followed by half a year CK.

**FIGURE 4:**
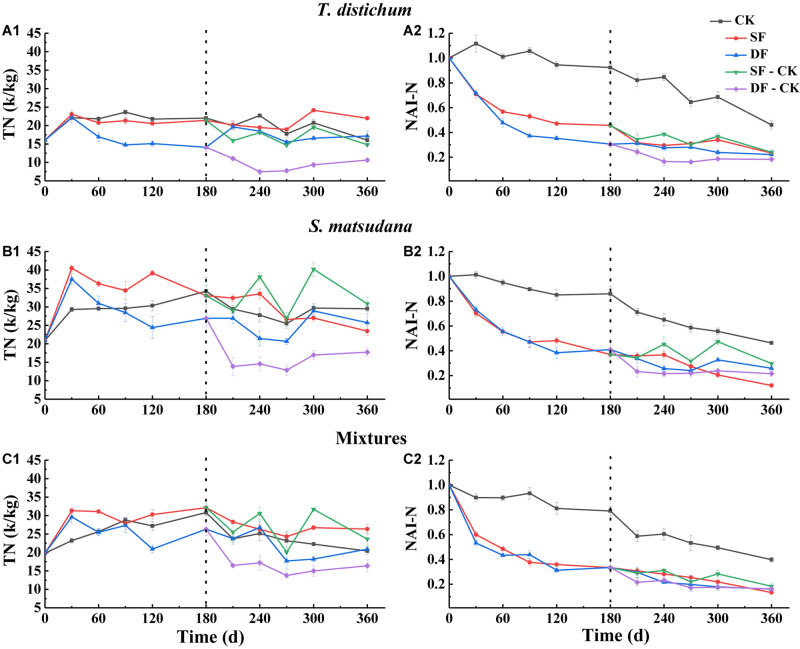
The variation of total nitrogen concentration **(A1–C1)** and NAI-N index **(A2–C2)** during the process of foliage decomposition. CK, unflooded; SF, shallow flooding; DF, deep flooding; SF-CK, first half a year SF followed by half a year CK; DF-CK, first half a year DF followed by half a year CK.

**FIGURE 5:**
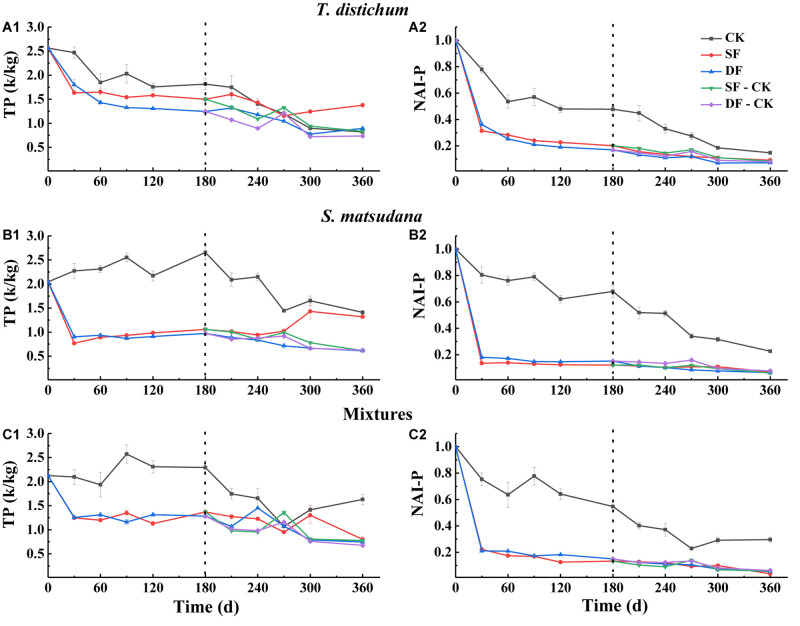
The variation of total phosphorus concentration **(A1–C1)** and NAI-P index **(A2–C2)** during the process of foliage decomposition. CK, unflooded; SF, shallow flooding; DF, deep flooding; SF-CK, first half a year SF followed by half a year CK; DF-CK, first half a year DF followed by half a year CK.

The change in the content of the leaf nutrients can only explain the proportion of a certain element in the mass remaining during its process of decomposition; it cannot directly reflect the loss of nutrients during the process of decomposition. Therefore, we expressed the degree of nutrient loss as the percentage of the net nutrient content to the initial amount at each stage of decomposition, which is characterized by the accumulation index NAI. An NAI < 1 or >1 means that the nutrients have been released or accumulated during the process of foliage decomposition, respectively. Nutrient dynamics and NAI varied among different species and water treatments (Figures 3–5).

During the entire process of decomposition, the TOC of each sample exhibited an overall decreasing trend (Figures 3A1–C1), the NAI-C of each foliage type under all experimental treatments declined continuously (Figures 3A2–C2), and the NAI-C was always less than 1. The NAI-C of each foliage type was the largest when decomposed in the CK environment (Figures 3A2–C2). When decomposed in the SF environment, the NAI-C of *S. matsudana* was the lowest (8.13%), and in the DF environment, the NAI-C of the mixtures was the lowest (14.31%) at the end of decomposition (*p* < 0.05). When decomposed in the SF-CK and DF-CK environments, the NAI-C of the mixtures was consistent with the flooding environment of the DF, which was the lowest, at the end of decomposition (9.32 and 9.29%, *p* < 0.05).

The dynamics of N and NAI-N dynamics during the decomposition of all foliage types are shown in Figure 4. Throughout the decomposition process, the N changed dynamically with the decomposition period (Figures 4A1–C1), while the NAI-N gradually decreased (Figure 4A2–C2); the rate of decrease in all foliage types was significantly faster than in the CK environment, and the NAI-N was less than 1 (except for the 30 and 90 days of *T. distichum* in CK, the 30 days of *S. matsudana* in the CK, the NAI-N was greater than 1). When decomposed in the CK environment, the NAI-N of all foliage types was significantly higher than that in the continuous flooding or flooded-to-unflooded hydrological process (Figures 4A2–C2). When decomposed in the SF environment, the NAI-N of *S. matsudana* (12.02%) was the lowest, while in the DF, SF-CK, and DF-CK treatments, the mixtures (16.01, 18.38, and 16.27%, respectively, *p* < 0.05) of the NAI-N were the lowest.

The P and NAI-P dynamics during the process of decomposition of all foliage types are shown in Figure 5. Except for the CK, the P decreased rapidly at the beginning of decomposition (Figures 5A1–C1). Similarly, NAI-P also declined the fastest in the first 30 days (Figures 5A2–C2); during the decomposition process, no matter what type of foliage, the NAI-P of each treatment was less than 1. The NAI-P of each foliage type under the CK environment was significantly higher than those of other environments (*p* < 0.05); the NAI-P of each foliage type was very closed (Figures 5A2–C2). Except for CK, the NAI-P of the mixed species was the lowest in SF, DF, SF-CK, and DF-CK water treatments, which was 3.78, 5.38, 5.69, and 6.23%, respectively (*p* < 0.05).

## Discussion

### Influence of Hydrological Regime on Foliage Decomposition

Traditionally, foliage decomposition has been roughly divided into three distinct processes: leaching, microbial action, and invertebrate feeding (Berg and McClaugherty, 2003; Hättenschwiler, 2005). Leaching mainly occurs in the early stage of decomposition. In aquatic ecosystems, leaching is more prominent because water is the main factor limiting the decomposition of land (Swarnalatha and Reddy, 2011; Wallis and Raulings, 2011; Sun et al., 2012). Flooding can accelerate early leaching and physical fragmentation during foliage decomposition (Wright et al., 2013; Whitworth et al., 2014; Chen et al., 2020b), which affects the ability of microorganisms to participate in decomposition and affects the foliage utilization rate by decomposers (Beth et al., 2012; Xie et al., 2016; Xiao et al., 2019). Litter can lose as much as 30% of its original mass during the initial leaching process, but there are obvious differences between species (Graça et al., 2005). In our study, at the beginning of decomposition (30 days), all foliage types lost as much as over 40% of their mass (Figure 2). Xiao et al. (2017) also found that in a soaking environment of the TGR, the mass loss peaked during the first month of decomposition. This may have been due to the rapid leaching of soluble components, leading to decreased leaf quality in the early stages of decomposition. As the decomposition duration increased, the nutrients that were easily lost were gradually decomposed. Simultaneously, the hard-to-decompose substances (such as cellulose and lignin) would be left, the proportion gradually increased, and the decomposition rate gradually slowed (Figure 2). However, in the second half of the decomposition period (180–360 days), even after being decomposed in the water for half a year, the soluble components had already been basically washed out, and the decomposition rate of each foliage type decomposed in dry conditions was still significantly lower than that in a continuously flooded environment (Table 3). This also confirmed that moisture is an important cause of foliage decomposition, whether it is applied at the beginning of decomposition or after a period of time. Similar to the findings of previous research on the acceleration of litter decomposition by flooding (Lecerf et al., 2007; Wallis and Raulings, 2011; Yue et al., 2016; Xiao et al., 2017; Yu et al., 2020), after the long-term immersion of litter in the water, not only during the early stage of decomposition, the strong leaching effect accelerated the rapid decomposition of soluble substances, but the strong mechanical breaking effect generated by the movement of water and debris would further affect the entire decomposition process (Lecerf et al., 2007; Zhang et al., 2015; Yue et al., 2016). In addition, aquatic microorganisms and invertebrates also play an important role in the process of foliage decomposition (Leroy and Marks, 2006; Tiegs et al., 2013). Fungi are the main decomposers in a flooded environment (Fonseca et al., 2013), and fungal biomass will increase significantly under flooding (Wright et al., 2013), which may also be the reason for the accelerated decomposition of leaves under flooding (Xie et al., 2016). Furthermore, relatively stable water temperatures also provide a favorable environment for biological activities to contribute to foliage decomposition (Table 1).

In our study, the effects of experimental treatment and decomposition time were also significant on the foliage remaining (*p* < 0.001; Table 2). Some studies have shown that foliage decomposition was closely related to the decomposed microenvironment, such as hydrological regime, the depth of flooding, and other abiotic factors, which can directly or indirectly affect the decomposition and nutrient release (Sun et al., 2012; Vivian et al., 2014; Casco et al., 2016; Xie et al., 2016; Yu et al., 2020). The decomposition rate of each foliage type in the SF environment during the first half of the year and the second half of the year was higher than that in the DF environment (except for the SF of *T. distichum* in the first half of the year) (Table 3). One of the reasons may be that the soluble organic matter in the SF had a higher leaching rate and attracted stronger microbial activity; under flooding environment, fungi can change the composition of their functional groups from terrestrial fungi to aquatic filamentous fungi in an area adapted to an anoxic environment, resulting in higher decomposition rates (Wallis and Raulings, 2011; Peltoniemi et al., 2012). Some previous studies have reached similar conclusions. Sun et al. (2012), studied *Calamagrostis angustifolia* litter decomposition in perennial flooding with different water depths and seasonal flooding; the results showed that the decomposition rates of a perennial flooding regime were much higher, and the shallowest perennial flooding had the fastest decomposition. Xie et al. (2016) also found that after 210 days of incubation, the decomposition rate of shallow flooding (5 cm) was greater than that of deep flooding (80 cm) because shallow submergence could stimulate the degradation of labile leaf litter.

In addition, the decomposition of foliage was also regulated by environmental factors (Table 4). In the first half of the decomposition period, regardless of the experimental treatment (CK, SF, and DF), the temperature and the mass remaining of each foliage type was significantly positively correlated; the electrical conductivity, pH, and dissolved oxygen were significantly negative with the mass remaining of the leaves in the SF and DF environment. The temperature of the SF environment was higher than that of the DF environment (Table 1). A higher temperature can increase the vitality of the fungus associated with decomposition (Fernandes et al., 2014; Martínez et al., 2014; Duarte et al., 2016). This promotion effect could occur in both high and low nutrient water bodies (Ferreira and Chauvet, 2011). It may be another important factor that affects the faster decomposition under the SF environment. Additionally, the level of dissolved oxygen was also higher in SF than that in DF, and the conductivity was low (Table 1). These environmental changes were also conducive to the growth and reproduction of microbial decomposers (Fernandes et al., 2014; Duarte et al., 2016). As the water depth changes, the redox conditions and light intensity will change, which may further influence the activity of decomposing microorganisms and, thus, the foliage decomposition (Wallis and Raulings, 2011; Xie et al., 2016). Therefore, a change in the depth of flooding, hydrological regime, temperature, dissolved oxygen, and other environmental factors can change the microenvironment and vary the rate of decomposition. In addition, the rate of decomposition may also be related to the difference in foliage quality between different foliage types (Table 6). All of these factors clearly showed that the foliage decomposition under the special hydrological dynamics in the reservoir area was conducive to material cycling in the reservoir area and played an important role in the material energy cycle of the entire riparian ecosystem.

**TABLE 6:** Initial foliar chemical characteristics of *Taxodium distichum* (A), *Salix matsudana* (B), and their mixtures (C).

**Foliage type**	**C (g⋅kg^–1^)**	**N (g⋅kg^–1^)**	**P (g⋅kg^–1^)**
A	470.38 ± 2.82a	16.05 ± 0.13c	2.56 ± 0.03a
B	423.85 ± 0.34c	20.90 ± 0.20a	2.05 ± 0.03b
C	450.05 ± 0.84b	19.78 ± 0.36b	2.13 ± 0.03b

*Different lowercase letters indicate a significant difference between species for the same variable at P < 0.05*.

In addition, we found that in the first half of the decomposition period, the decomposition rate of the mixed species in the three decomposition environments of CK, SF, and DF was the fastest. In the second half of the decomposition period, except the CK and SF environments, the decomposition rate of the mixed species in the DF, SF-CK, and DF-CK environments were also the fastest (Table 3). This means that during the decomposition process of continuous flooding in the first half of the year and the dry conditions in the second half of the year, the leaves of mixtures of *T. distichum* and *S. matsudana* accelerated the decomposition process (Figure 2). It was generally believed that the mixing of coniferous and broad-leaved species would accelerate decomposition in the terrestrial environment (Gessner et al., 2010; Zeng et al., 2018). In our study, the mixed-species decomposition in the flooded environment was similar to that of the terrestrial environment, but it happened earlier and was more intense; it accelerated the decomposition in both continuous flooding and flooded-to-unflooded hydrological process (Figure 2 and Table 3). This might be because the high nutrient content of *S. matsudana* promotes nutrient availability for *T. distichum*, which has relatively low nutrient content initially and causes the decomposition of *T. distichum* to accelerate during the process of decomposition (Table 6). After *S. matsudana*, which had a high N concentration, was mixed with the low initial N of *T. distichum*, different concentrations of N could be transported between the mixed leaf litter through fungal hyphae or leaching (Schimel and Hättenschwiler, 2007). The high N concentration will transfer N into foliage types with low substrate mass, resulting in an increased decomposition rate. In addition, the mixtures may promote the rapid colonization of decomposer communities, thereby accelerating the mass loss of slowly processed recalcitrant leaves (Ostrofsky, 2007). Our research found that the decomposition of the mixtures in some experimental treatments accelerated the decomposition rate, which may be caused by the above-combined effects.

### Influence of Hydrological Regime on Foliage Nutrient Release

In our study, decomposition time, experimental treatment, and their interactions had significant effects on nutrient remaining from each foliage type (*p* < 0.001; Table 2). In a flooded environment decomposition process, the concentrations of nutrients and the microbial biomass of foliage were lower when compared with the unflooded environment, indicating that the transportation and circulation of water flow may cause a rapid decrease in nutrient concentrations in a flooded environment (Kominoski et al., 2007; Lecerf et al., 2007). In the first half of the flooded decomposition period, the release rates of TOC, TN, and TP in the SF and DF environments were also significantly greater than that in CK (Table 5). Thus, the nutrients in each foliage type have been found to be more stable in unflooded environments than in flooded environments. This was similar to the study of Shieh et al. (2008), who found that the litter of *Machilus thunbergii*, *Schefflera octophylla*, and *Ficus erecta* released nutrients more rapidly when submerged in the subtropical rivers in northern Taiwan, which was related to their initial nutrient concentration. In addition, the NAI-C, NAI-N, and NAI-P in each foliage type were less than one at the end of decomposition, and much less than CK; the nutrients of the three foliage types were in a net release state (Figures 3–5). This further showed that the long-term flooding in the riparian zone of the TGR area could promote the release of nutrients from leaves of reforestation tree species to a certain extent.

The effects of the flooded environment on the nutrients remaining may be related to the characteristics of the leaves, microenvironmental conditions of decomposition, and the selective feeding of aquatic decomposers among different species (Xie et al., 2016; Xiao et al., 2019). Among them, the concentrations of TN and TP in the leaves were important factors that affect decomposition; changes in their concentrations will alter the release of nutrients (Ball et al., 2009; Berglund et al., 2013; Xiao et al., 2017). The present study found that, in the first half of the decomposition period, the release rates of TOC and TN of the mixtures were the fastest in the CK and SF environments (Table 5). In addition, the mixtures had the lowest of NAI-N (Figure 4), which showed that the C and N cycles as well as the nutrient transfer in the mixtures were very dynamic and could promote the decomposition and energy flow of matter. Besides, previous soaking experiments have shown that the decay and decomposition of plants in the TGR cannot only release large amounts of TN but also release large amounts of TP (Xiao et al., 2017). The form of P in leaves exists as a biologically active element, making it easier to be released (Schindler and Gessner, 2009; Jacobson et al., 2011). During the decomposition process, it can be released abruptly into the water (Sommaruga et al., 1993). Fraser et al. (2004) reported that P could be released through plant migration and leaching because it exists in the form of phosphate anions or compounds. Our study supported this finding; the release of TP was much faster than that of TOC and TN in all foliage types (Table 5). For the TP release rate of the SF environment in the first half of the year, as well as the SF, DF, SF-CK, and DF-CK environments in the second half of the year, the mixed species were all the highest. These showed that the release of nutrients from the reforestation tree species, especially the mixed-species samples, may have accelerated the nutrient cycle and energy flow in the riparian zone of the TGR.

In addition to leaf characteristics, other environmental factors and microbial action that affect the decomposition may also indirectly influence the release of nutrients (Gessner et al., 2010; Zanne et al., 2015; Bradford et al., 2016). For example, oxygen levels differed between aquatic and terrestrial systems, and the dissolved oxygen level also varied with different water depths (Table 1), which would lead to different levels of decomposer activity in these two systems. Besides, although terrestrial litter generally has higher microbial biomass, stream fungi were more efficient at decomposing leaves than terrestrial fungi (Gessner and Chauvet, 1994; Hieber and Gessner, 2002). However, terrestrial microorganisms are more capable of immobilizing and retaining the nutrients of litter in leaves because they are less likely to be restricted by the hydrological flow. Our research also confirmed this; the amount of nutrients remaining of the samples decomposed in CK environment was significantly greater than that in continuous flooding and flooded-to-unflooded hydrological processes (Table 5). In short, the differences in the nutrient release under different hydrological regimes are mainly caused by the specific foliage traits, environmental conditions, and selective aquatic decomposition of different species (Gessner et al., 2010; Jabiol and Chauvet, 2012; Fernandes et al., 2014).

## Conclusion

In summary, different hydrological regimes could promote the mass loss and nutrient release of the leaves of the dominant afforestation tree species in the riparian zone of the TGR area. The hydrological regime, decomposition time, foliage types, and their interactions had a very significant effect on foliage decomposition and nutrient release. Especially for the mixed species, the decomposition rate of all experimental treatments (CK, SF, and DF) in the first half of the year was faster than that of a single species, and the decomposition rate of DF, SF-CK, and DF-CK environments in the second half of the year was also faster than that of a single species. Both hydrological processes and different depths of flooding accelerate the material circulation and energy flow in the reservoir area. In addition, the experiment did not test the factors influencing microorganisms and only made speculations using relevant research. It should be followed up in future experimental studies.

## Data Availability Statement

The raw data supporting the conclusions of this article will be made available by the authors, without undue reservation.

## Author Contributions

CL, ZC, and MA conceived the study, designed the experiments, and supervised the entire study. ZC, CW, and XC performed the experiments. CL and ZC wrote the manuscript. All authors contributed to the article and approved the submitted version.

## Conflict of Interest

The authors declare that the research was conducted in the absence of any commercial or financial relationships that could be construed as a potential conflict of interest.
